# Editorial: Human Milk in the Feeding of Preterm Infants: Established and Debated Aspects

**DOI:** 10.3389/fped.2020.00378

**Published:** 2020-08-21

**Authors:** Guido E. Moro, Sertac Arslanoglu

**Affiliations:** ^1^European Milk Bank Association (EMBA), Milan, Italy; ^2^Associazione Italiana Banche del Latte Umano Donato (AIBLUD), Milan, Italy; ^3^Division of Neonatology, Department of Pediatrics, Istanbul Medeniyet University, Istanbul, Turkey

**Keywords:** breastfeeding, human milk, donor human milk, human milk banks, preterm infant feeding, human milk fortification

Evidence indicates that human milk (HM) is the best source of nutrition not only for term but also for preterm infants conferring health benefits both in the short and long-term (1–3). Thus, in the last few decades, HM has been identified as the normative standard for preterm infant feeding by the scientific health authorities (1, 2, 4). Human milk reduces the chance of developing necrotizing enterocolitis (NEC), sepsis, and other infections, as well as bronchopulmonary dysplasia (BPD) and severe retinopathy. It has also been shown to decrease the risk of death and improve long-term neurocognitive development and cardiovascular health outcome. Yet, HM does not provide sufficient nutrients to very low birth weight (VLBW) infants when fed at the usual feeding volumes. Therefore, it should be supplemented (fortified) with nutrients in short supply, particularly protein, calcium, and phosphate to meet the high requirements of this group of tiny preterm infants. During the last decade, optimization of HM fortification, mainly individualization, and the quality of the fortifiers have been the topics of discussion, aiming to improve the clinical outcomes of these babies (1, 2, 4).

Processing HM in human milk banks may alter the quality of the milk. Holder pasteurization (62.5°C for 30 min) is currently the procedure recommended by all human milk banks to ensure the microbiological safety of HM, but alternative methods are under investigation (1, 4).

This Research Topic is aimed at collecting papers suitable to improve our knowledge and understanding on HM composition, HM fortification, processing and handling, and feeding practices with associated clinical outcomes.

In this special e-collection there are 25 papers covering the above mentioned aspects.

Processing of human milk has been the most evaluated aspect. Nine papers out of 25 (36%) were related to this topic, which represents one of the most important steps in the operative procedures of Human Milk Banks (HMBs). Currently, a pasteurization process performed at a temperature of 62.5°C for 30 min, which is known as the Holder pasteurization (HoP), is recommended in all the international guidelines for the inactivation of viral and bacteriological agents present in donor human milk (DHM). However, HoP affects some of the nutritional and biological properties of fresh human milk. Data from Bertino et al. show that HoP affects negatively oxidative stress markers to variable degrees. Also cytokines are negatively affected by HoP, and their concentrations decline following HoP, with the exception of IL-8 that is preserved (89%) after pasteurization (Giorgi et al.).

Due to these limitations, there is the need to evaluate alternative processing methods able to better preserve the bioactivity of a higher number of HM components in order to improve the nutritional and immunological quality of DHM. Research on some of these technologies, like High-Temperature-Short-Time (HTST), High Pressure Processing (HPP) and ultraviolet-C (UV-C) irradiation are extremely promising but still in a phase of evaluation. HTST seems to be better than HoP at preserving the HM antioxidant potential, lactoferrin content and structure, and some cytokines which describe the advantages of this methodology (Moro et al.). The group of Lembo presented data showing that, unlike the HoP, HTST preserved the inhibitory activity against cytomegalovirus, respiratory syncytial virus, rotavirus, and herpes simplex virus type 2 (Donalisio et al.). Klotz et al. confirm the antiviral activity of HTST against cytomegalovirus, but show that this technology is less effective than HoP in bacterial count reduction. Two HTST pasteurizers have recently been specifically designed and validated for human milk processing (Moro et al.).

Also high pressure processing (HPP) is considered a promising alternative to thermal pasteurization of human milk. HPP leads to preservation of adipokines, growth factor, lactoferrin, and IgG much better than HoP (Wesolowska et al.). Demazeau et al. have recently optimized the operational parameters of HPP and this has allowed the inactivation of B. cereus spores while preserving the bioactive factors. The main obstacle to the use of HPP in human milk treatment, is the scaling down of the equipment and the investment and operating costs (Moro et al.). At the moment, the most practical solution to improve processing of human milk seems to be an optimization of HoP including an accurate control of the heating phase, with a quality control of the pasteurizer performed regularly, at least once a year (Buffin et al.). Moreover, since HoP is the most frequently used technique, it should be evaluated, as a part of the optimization process, whether a temperature below 62°C could be utilized in terms of improved preservation of fresh human milk properties without compromising the microbiological safety (Gayà and Calvo).

Mother's own milk (MOM) is the first choice for premature infant feeding. When MOM is not available or is insufficient, DHM from an established human milk bank represents the best alternative, with well-documented advantages compared to formulas derived from bovine milk (1). In this Research Topic, five papers refer to the advantages of HM when utilized in feeding preterm infants. Boquien et al. give a comprehensive overview regarding the composition of human milk and its correlation with infant growth and neurodevelopment, while Ziegler addresses the art of feeding the preterm infants with human milk in their early days of life. The main clinical advantage deriving from utilization of human milk in feeding preterm infants is prevention of NEC. New combined data from *in vitro* tissue culture models, *in vivo* preclinical studies in animal models, and human mother-infant cohort studies support the hypothesis that some specific human milk oligosaccharides contribute to the beneficial effects of human milk feeding in reducing NEC (Bode). The first systematic review investigating the effects of MOM on BPD confirms the beneficial effects of mother's milk, at least when used as an exclusive diet (Villamor-Martínez et al.).

Above protection from NEC and BPD, human milk is able to modulate the quality of growth in preterm infants, with higher fat-free mass percentage when intakes >50% fortified human milk are obtained (Piemontese et al.).

Because of the extremely high nutritional requirements of premature infants, human milk must be fortified with nutrients, particularly with protein and minerals, to ensure optimal nutrient intake and adequate growth. Best fortification strategies, as well as the “optimal” composition of fortifiers are still objects of research. In this e-book there are 3 papers related to this topic. The EMBA Working Group on Fortification of Human Milk encourages the use of “individualized fortification” to optimize nutrient intake. The quality and source of human milk fortifiers constitute another important aspect. There is work looking at human milk derived fortifiers, but it is still too early to draw precise conclusions about their use. Many other practical recommendations on fortification of HM can be found in this article (Arslanoglu et al.). Fortification of HM increases its osmolality, and a high value of osmolality is associated with an increased risk of NEC. The study of Kreins et al. shows very clearly that osmolality increases significantly immediately after fortification, depending on the type of fortifier used. A practical aspect to keep in mind is that, as most of the increase in osmolality occurs immediately, bedside fortification is not useful to prevent the increase in osmolality (Kreins et al.).

Protein intake is the limiting factor for the regular growth of preterm infants fed fortified human milk during the hospital stay and after discharge. There are very few long term follow-up studies comparing standard protein intake vs. high protein intake in VLBW infants. Mariani et al. evaluated the effects of two different protein intakes (Standard Protein Intake: 3.5 g/kg/day; Aggressive Protein Intake: 4.5–5.0 g/kg/day) on feeding tolerance, hospital growth, anthropometric data and psychomotor outcome up to 24 months corrected age in extremely low birth weight infants (ELBW; birth weight <1,000 g) fed fortified human milk. The infants receiving the high protein intake performed significantly better for all the parameters evaluated both during and after hospitalization, showing short and long term benefits in terms of growth and neurodevelopment (Mariani et al.).

The feeding of human milk to preterm infants is typically much more complicated than the mere act of breastfeeding. The discussion of safe HM handling and administration has extended beyond infection prevention to comprise other critical errors like misadministration, fortification errors, and the feeding of expired milk. A survey performed by Klotz et al. related to handling of HM in neonatal units from Germany, Switzerland, and Austria clearly shows a wide variability in most aspects of HM handling. To overcome this variability, Steele in her mini-review article summarizes current published best practices for the handling of HM for preterm infants within the hospital setting. Emphasis is focused on the use of aseptic technique, and the use of technology to prevent misadministration of HM and fortification errors as well as for tracking of expiration dates and lot numbers (Steele).

At the end of this quick ride through the main aspects of human milk in the feeding of preterm infants, there are still two papers that deserve a mention. The first is the paper from Davanzo where controversies in breastfeeding are discussed. A useful differentiation is done between “contraindication” and “obstacle.” Failure to distinguish between these two conditions confuses the new mothers and their families, and engenders misconceptions among health professionals. The list of “true contraindications” to breastfeeding is short and clearly stated, and it is presented in an easily understandable table (Davanzo).

The second paper is produced by the Guideline Working Group of the EMBA. MOM is the first choice in preterm infants feeding, and strong efforts should be made to promote lactation. When mother's milk is not available, donor human milk is the preferred choice and it should be provided by a well-established HMB (1). There are at present several international available guidelines for the establishment and operation of HMBs, but there are no Europe-wide guidelines. So, one of the clear objective of the EMBA has been to develop at least Europe-wide recommendations. The processes and practices within human milk banking do not lend themselves to randomized controlled trials and there are few meta-analyses or systematic reviews available to refer to. In the absence of these, expert opinion is required. This is the reason why this Working Group decided to write recommendations and not guidelines for HMBs in Europe. The pragmatic approach to the items where differences could not be resolved through reference to published research, resulted in a practical “guide” to cope with the most important issues related to the world of human milk banking.

In summary, the results of the above mentioned studies and reviews represent an enormous amount of new relevant data on the composition, fortification, processing and advantages of HM when utilized in preterm infants feeding. Despite all the existing literature and evidence related to this extremely important topic, the papers published in this e-book clearly show that there are still many aspects to be clarified and understood in the fascinating world of HM and preterm infant nutrition. After reading this book, some topics as the composition of HM, fortification of HM when utilized for feeding VLBW infants, handling of HM in neonatal units, feeding practices in preterm infants, and processing of DHM, will appear more clear to the reader and reinforce the belief that HM represents the best food in feeding all neonates, including preterm infants.

## Author Contributions

GM wrote the introduction and the conclusion. SA wrote the central part with comments to the cited papers and references. All authors contributed to the article and approved the submitted version.

## Conflict of Interest

The authors declare that the research was conducted in the absence of any commercial or financial relationships that could be construed as a potential conflict of interest.
